# Three-dimensional nanomagnetism

**DOI:** 10.1038/ncomms15756

**Published:** 2017-06-09

**Authors:** Amalio Fernández-Pacheco, Robert Streubel, Olivier Fruchart, Riccardo Hertel, Peter Fischer, Russell P. Cowburn

**Affiliations:** 1 Cavendish Laboratory, University of Cambridge, JJ Thomson Avenue, Cambridge CB3 0HE, UK; 2 Division of Materials Sciences, Lawrence Berkeley National Laboratory, Berkeley, California 94720, USA; 3 Univ. Grenoble Alpes, CNRS, CEA, Grenoble INP, INAC, SPINTEC, F-38000 Grenoble, France; 4 Université de Strasbourg, CNRS, Institut de Physique et Chimie des Matériaux de Strasbourg, UMR 7504, Department of Magnetic Objects on the Nanoscale, F-67000 Strasbourg, France; 5 Department of Physics, UC Santa Cruz, Santa Cruz, California 95064, USA

## Abstract

Magnetic nanostructures are being developed for use in many aspects of our daily life, spanning areas such as data storage, sensing and biomedicine. Whereas patterned nanomagnets are traditionally two-dimensional planar structures, recent work is expanding nanomagnetism into three dimensions; a move triggered by the advance of unconventional synthesis methods and the discovery of new magnetic effects. In three-dimensional nanomagnets more complex magnetic configurations become possible, many with unprecedented properties. Here we review the creation of these structures and their implications for the emergence of new physics, the development of instrumentation and computational methods, and exploitation in numerous applications.

## Abstract (web summary)

Nanoscale magnetic devices play a key role in modern technologies but current applications involve only 2D structures like magnetic discs. Here the authors review recent progress in the fabrication and understanding of 3D magnetic nanostructures, enabling more diverse functionalities.

Nanomagnetism, the scientific field dedicated to the study of nanoscale magnetic objects, has undergone an explosion of activity over the last few decades, driven by fascinating discoveries such as the interaction of magnetization with spin currents (the area of spintronics)^1^,^2^ and a wide range of real-world applications^3^,^4^,^5^,^6^,^7^. For example, since both the storage and sensing parts of hard disk drives use nanomagnetic structures, the development of nanomagnetism has been a key factor in the vast recent improvements in computer performance and the development of cloud computing. With the exception of some self-assembled systems^8^,^9^, nanomagnetism has been mostly confined to two dimensions. In two-dimensional (2D) patterned planar single- or multi-layered magnetic structures, the thickness is of the order of some characteristic magnetic length-scale. This simple geometry leads to monodomain magnetic states in the vertical direction, restricting functionality to the substrate plane (Fig. 1b). In these cases, complex and useful magnetic behaviour is nevertheless possible by exploiting interfacial effects between layers.

The maturity now reached in this field^10^, together with the need for low energy technologies with new functionalities^11^ and the advent of advanced chemical and self-assembly synthesis techniques capable of growing non-planar nano-objects, make the expansion of nanomagnetism into three dimensions possible (Fig. 1a). In this new paradigm, spin configurations and capabilities extend not only in the plane but also into the vertical direction, and more complex, hierarchical systems leading to new effects can be designed. In this article, we review the state-of-the-art of three-dimensional (3D) nanomagnetism: the new physics associated with different types of 3D magnetic nanostructures, the most promising synthesis and characterization techniques available to create (Box 1) and probe (Box 2 and Box 3) these systems, and the latest advances in computational methods for their modelling (Box 4). We also highlight some of the major challenges still associated with these studies, and the great potential of this field to impact areas such as sensing, data storage, nanoelectronics, the Internet-of-Things and medical biology.

## Emerging physics of 3D nanomagnetism

### New types of domain walls

The extension of 2D nanostructures into the 3D world brings with it the emergence of unconventional spin textures, where novel physical effects comprising geometry, topology and chirality are involved. A paradigmatic example regards domain walls (DWs) in magnetic nanowires (NWs). NWs are subject to extensive investigation in nanomagnetism because of their potential use as DW conduits for memory and sensing applications^12^,^13^,^14^. Considering in detail NWs formed by soft magnetic materials, magnetization tends to be aligned parallel to their long direction, due to shape anisotropy. In this case, DWs are of either head-to-head or tail-to-tail type, holding a magnetostatic charge 2*SM*_s_ (with *S* the NW section and *M*_s_ the spontaneous magnetization of the material), irrespective of the spin texture within the wall. This gives rise to magnetostatic energy, such that competition with exchange determines a complex panorama of DW types, in a manner depending on dimensionality and strip size. The size is compared with the characteristic dipolar exchange length *Δ*_d_=(2*A*/*μ*_0_*M*_s_^2^)^1/2^ (ref. 15), where *A* is the exchange stiffness of the material. We first describe their static features, before highlighting some peculiar aspects of their dynamics.

**Figure 2:**
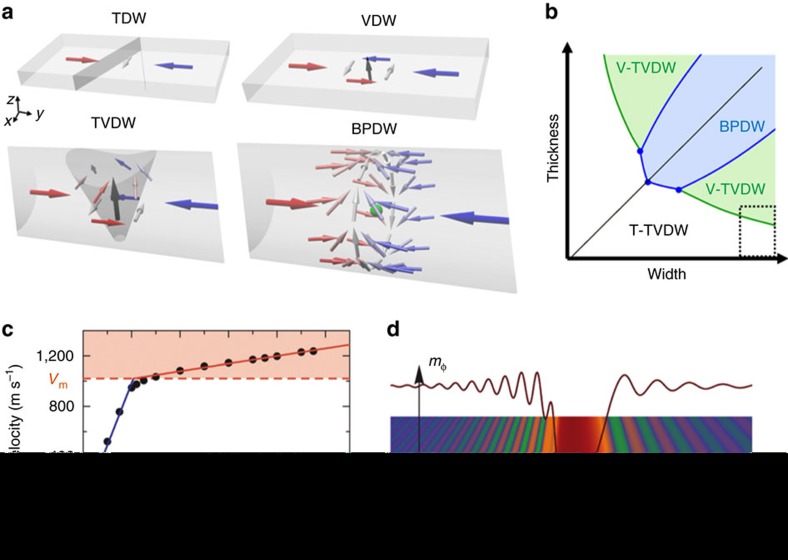
New physical effects for DWs in 3D NWs and NTs. (**a**) Types of DWs in nanostrips (transverse: TDW and vortex: VDW) and in 3D NWs (transverse-vortex: TVDW and Bloch-point: BPDW). The shaded areas represent the wall shape for the TDW and TVDW. BPDW: the green sphere represents a Bloch point. (**b**) Thickness-versus-width energy schematic phase diagram for DWs in NWs. These dimensions are relative to the dipolar exchange length in wires made of soft magnetic materials. In the white and green regions, TVDWs are typically observed, whereas in the blue area, BPDWs are energetically favourable. The region marked by dashed line represents the typical area referring to nanostrips, with widths much larger than thickness, where either TDW or VDW are observed. (**c**) Velocity of a DW with axial vortex configuration propagating in a NT as a function of external magnetic fields. Very high speeds are reached without experiencing a Walker breakdown. Two regimes with different mobilities are observed: the usual motion, described as Walker, and Magnonic (red region), above a critical velocity *v*_m_. The kink at the transition shows the change of mobility (effective mass) of the wall. (**d**) Snapshot of a simulation in the Magnonic regime, showing strong spin wave emission during DW propagation. The figure shows the azimuthal component of the magnetization (*m*_ϕ_) in an unrolled NT, for an easier visualization. Panels (**c**) and (**d**) are reproduced from ref. 36 with the permission of AIP Publishing.

2D NWs (from now on denoted nanostrips to distinguish them from their 3D counterparts) have been widely studied in theory, simulation and experiments, because of their ease of fabrication and integration into devices. These are patterned with standard top–down lithography methods from thin films, having widths much larger than *Δ*_d_ and thicknesses comparable or not much larger than a few times this length. Thus, any significant variation of magnetization across thickness is prohibitively expensive energetically, such that magnetization textures can be faithfully described by a 2D field of a 3D magnetization vector **M**(*x*,*y*). For those nanostrips with moderate widths, significant variation of magnetization across the nanostrip would also be energetically unfavourable. Therefore, such DWs may be described using a 1D model by **M**(*y*), where magnetization in the core of the wall is aligned transverse to the strip long axis *y*; this is the so-called transverse DW (TDW, see Fig. 2a). Obviously, the transverse component imparts additional dipolar energy due to the edge charges that are created. For sufficiently wider strips, this energy can be reduced by the emergence of a different type of magnetic boundary, the vortex DW (VDW): here, magnetization becomes more parallel to the strip edge by curling around a vortex with core perpendicular to its surface (Fig. 2a). These two types of DWs share a large metastability region in the phase space, consisting of thickness (*t*) versus width (*w*), around an iso-energy line, determined by simulations^16^ as *tw*≃61*Δ*_d_^2^.

To extend this already well understood phase diagram (see dashed region in Fig. 2b) to an arbitrary cross-section, the thickness of a nanostrip can be progressively increased towards a NW of square cross-section. DWs initially transverse and vortex in nature end up in magnetization textures with a tube of magnetization going through the wire along a transverse direction, *x* and *z* respectively. Remarkably, these two types of walls, characterized now by a full 3D magnetization field **M**(*x*,*y*,*z*), are identical upon rotation by *π*/2 around the wire axis. For large lateral wire dimensions, they display simultaneously transverse and vortex characteristics. They may, therefore, be denoted transverse-vortex domain walls (TVDWs)^15^, whereas the denotation of either transverse or vortex is sufficient in nanostrips (or *π*/2-rotated: very narrow and thick NWs), where one of these features is largely dominant (Fig. 2a). Being able to transform from one to the other using only continuous transformations from the flat nanostrips reveals that they share the same topology. This topological equivalence remains valid for any NW cross-sectional shape, for instance either square or circular.

Until now in our discussion, 3D NWs were considered as if the 2D DW magnetization was simply extruded along the third direction. However, considering 3D NWs from the start allows for the emergence of novel spin textures. The DWs so far described display two areas where magnetization is perpendicular to the wire surface: the entry and outlet points of the vortex core. Since magnetization pointing perpendicular to a surface implies an increase in magnetostatic energy, DW configurations with a more efficient magnetic flux closure (that is, with magnetization mostly parallel to the wire surface at any point) should be expected. Such a texture is possible for cylindrical symmetry, leading to a curling of the magnetization parallel to the wire axis. For a solid cylindrical NW, and with these boundary conditions, the continuity of a vector field of uniform magnitude becomes impossible^17^. Hence, there must exist one point where **M** cancels, that is, a local singularity where ferromagnetism is quenched. This very peculiar object, called a Bloch point, was theorised decades ago, based on similar external boundary conditions in bubble media^18^,^19^. It refers to a micromagnetic object of zero dimensions (0D); it is the counterpart of Bloch walls as 2D objects separating 3D domains. This wall, shown in Fig. 2a, is characterized by an axial vortex, that is, its axis is parallel to the wire, and with the Bloch point lying on the axis. It can accordingly be named a Bloch point DW (BPDW)^20^. Its existence was first predicted by means of micromagnetic simulations^21^,^22^. Taking these considerations into account, a general thickness-versus-width phase diagram reflecting the energetics of TVDWs and BPDWs in 3D NWs can be constructed (Fig. 2b). In the white and green regions of the diagram, the TVDW is the DW ground state, with either transverse or vortex features, respectively, becoming more evident for each area. The blue region, on the contrary, refers to the parameter space where the BPDW is the ground state, which occurs for lateral dimensions typically above 7*Δ*_d_. It is worth noting that there exist large areas of this phase diagram where two or even three of these walls may coexist as (meta)stable states^15^. The first experimental imaging of TVDWs^23^,^24^ and BPDWs^24^ in NWs has recently been reported.

The above description of DWs in NWs illustrates characteristics of 3D spin textures displayed by other non-planar nanomagnetic objects. For instance, nanotubes (NTs) exhibit a rather similar phenomenology^25^, with just qualitative differences of the iso-energy lines, and notably with the absence of the Bloch points seen in the case of DWs with an axial vortex. The ends of elongated objects are also worthy of consideration, as they constitute the loci of nucleation for magnetization reversal. With the same physics (magnetostatics versus exchange) at play, these loci display end states called ‘C' or curling, which may be viewed as a fraction of a TDW or BPDW, respectively^22^.

Additionally, unlike planar surfaces, curved surfaces lack space inversion symmetry. This can be the source of remarkable effects^26^. For instance, micromagnetic simulations have shown that the surface curvature in cylindrical NTs and NWs can give rise to a chiral symmetry breaking that results in different properties, depending on the rotation sense of the magnetization. This effect resembles the Dzyaloshinskii–Moriya interaction (DMI)^26^. To address this phenomenon of curvature-induced DMI, a 3D theory has recently been proposed^27^, consisting of reducing magnetostatic effects to an effective anisotropy. Under this assumption, curvature and torsion in NWs and shells split up the existing exchange interaction into scalar Heisenberg exchange and two effective magnetic interactions, namely curvature-induced effective anisotropy (magnetization patterning) and curvature-induced effective DMI (chirality selection)^27^,^28^. Systematic studies of magnetic NTs have proven the existence of an exchange energy that manifests as an effective easy-axis anisotropy along the tube axis^25^,^29^, which induce a gap in the dispersion relation of magnonic spin excitations in NTs^30^. Remarkably, the strength of the curvature-induced chiral effects could be tuned mechanically by bending flexible magnetic nanomembranes^31^, thereby providing a new method to manipulate magnetic properties at the nanoscale.

### Dynamic effects

Dynamic magnetic properties may also differ significantly between 2D and 3D nanomagnets, as recently demonstrated in the case of DW propagation and NWs. The dynamic motion of DWs in NWs is of paramount importance for their exploitation in applications. This has led to a large amount of works in nanostrips in the last years^20^,^32^,^33^,^34^, where a complex scenario including several regimes of DW propagation, can be observed. In particular, DWs under external fields usually become unstable when propagating above a critical speed, an effect called Walker breakdown^35^. This effect originates from a torque exerted by the driving field on the DW. If this torque is sufficiently strong, the wall magnetic structure changes significantly in a periodic fashion, causing the motion to become oscillatory, as described by the Landau–Lifshitz–Gilbert equation^20^. Recent studies using micromagnetic simulations^36^,^37^ carried out in cylindrical NWs and NTs, reveal that DWs with an axial vortex configuration (BPDWs in the case of NWs) can propagate smoothly at very high speed (∼1 km s^−1^) when driven by an external magnetic field, overcoming the Walker breakdown^36^,^38^. Axial VDWs in NTs and NWs can surpass this limit due to the radial component of the magnetization^36^,^37^, caused by magnetostatic field distributions. This stability against the Walker breakdown in NTs and cylindrical NWs, described by micromagnetics, can also be understood in the context of the aforementioned chiral break of symmetry due to the surface curvature^36^. The effective dynamic-DMI counteracts this torque and thereby preserves the magnetic structure of the DW, even at large fields. Since this is a chiral effect, only one of the two possible vortex configurations of the DW is stabilised. These ultra-stable DWs can even reach the phase velocity of spin waves and thereby give rise to the Spin-Cherenkov Effect^36^ (Fig. 2c,d). This effect describes the spontaneous emission of spin waves occurring when a perturbation in a magnetic system moves faster than a threshold velocity, a phenomenon similar to sound waves generated by supersonic aircraft crossing the sound barrier. At these elevated velocities, DWs emit bichromatic spin waves of velocity-dependent wave length^36^.

Moreover, while the static structure of BPDWs in cylindrical NWs is now relatively well understood, the dynamic structure remains largely unexplored. This is because BPDWs are difficult to access experimentally and because the singularities of the magnetization field are characterized by a pronounced inhomogeneity of the magnetization that persists down to the atomistic scale, thereby making their simulation unreliable with standard micromagnetic codes. To resolve this problem, a hybrid atomistic-micromagnetic method has been recently developed with which the structure and the dynamics of BPDWs in NWs have been simulated at the atomic scale^39^, predicting a peculiar oscillation in the dynamics during field-driven motion^40^.

### 3D spin textures

**Figure 3:**
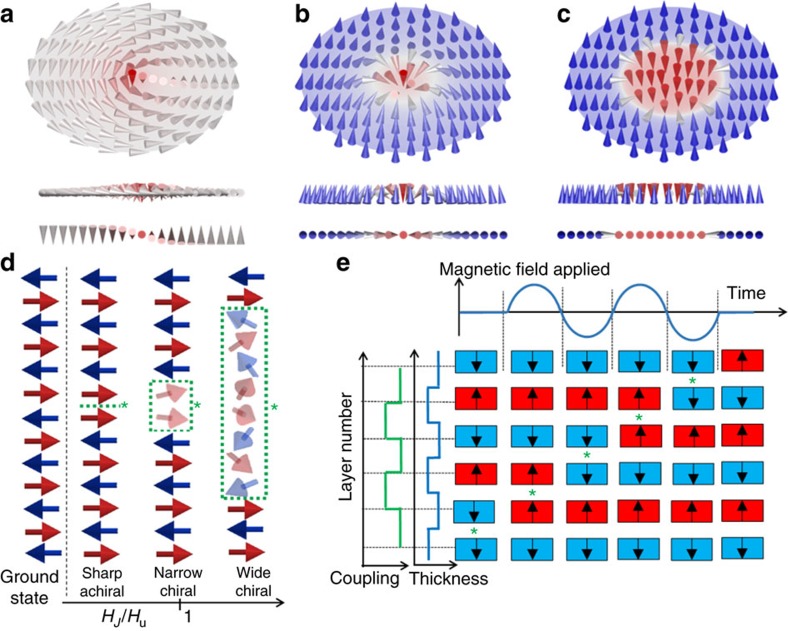
Three-dimensional spin textures. Schematics of (**a**) magnetic vortex, (**b**) (Neel) skyrmion and (**c**) chiral bubble, shown at different perspectives (oblique, side and top view). Whereas, the vortex nucleation is in general limited to nano- and micro-patterned planar magnets due to the interplay between Heisenberg exchange and magnetostatic contributions, skyrmions and chiral bubbles emerge in systems with broken inversion symmetry, for example, via antisymmetric Dzyaloshinskii–Moriya exchange interactions. (**d**) Macrospin model of an antiferromagnetic superlattice where the ground state corresponds to all spins antiparallel to each other. The introduction of a soliton divides the system in two anti-phases. The width and chirality (defined as the sense of rotation of the spins–clockwise or counter clockwise) of the soliton depends on the exchange (*H*_*J*_)/anisotropy (*H*_u_) field ratio of the system. The middle point and extension of the soliton in each case is marked by an asterisk and a green dashed rectangular area, respectively. Faded spins represent in-plane deviations from the easy axis. (**e**) Ratchet scheme for propagation of sharp achiral solitons. RKKY coupling and thickness change periodically between two values throughout the superlattice. The soliton, marked with an asterisk, moves synchronously under oscillating magnetic fields, propagating one step upwards every half field cycle.

3D magnetic spin textures with non-trivial topological charges (vortices, skyrmions and chiral bubbles) possess an exceptional stability under external perturbations against transitions into trivial states, for example, to collinear magnetization. Enhanced stability, particle-like properties and high susceptibility to electric, spin and heat currents, and magnonic excitations have put these chiral structures into the spotlight of fundamental sciences, as well as application-oriented research. A paradigmatic 3D spin texture is the magnetic vortex with its nanometric core magnetization (Fig. 3a). Experimentally observed for the first time almost two decades ago^41^, it is typically present in patterned thin films formed by soft magnetic materials. The spin structure is defined by both in-plane magnetic circulation and out-of-plane core polarity, with the core expanding laterally ∼10 nm^42^, as set by *Δ*_d_. This strong confinement has allowed the quantification of pinning site potentials in thin films by probing Barkhausen noise^43^, generate nano- and micro-oscillators with tuneable frequency^44^ and design of magnetic vortex memories^45^.

While the chirality of vortices (defined as the product of circulation and core polarity) and other objects such as Bloch points is intrinsically degenerate in common 2D nanomagnets, 3D geometries with inherent new types of interactions can break this symmetry. As mentioned above, this is the case of 3D curved nanomagnets, which reveal a chirality selection due to curvature-driven DMI^46^. Similarly, heterostructures formed by ultrathin magnetic thin films and heavy-element materials with large spin–orbit coupling, result in interfacial antisymmetric spin couplings due to spin-polarized scattering from conduction electrons in the heavy-element layer. This antisymmetric exchange interaction, referred to as interfacial DMI (IDMI)^47^, favours non-collinear chiral spin configurations. The competition of IDMI with standard symmetric exchange interactions (that is, Heisenberg and Ruderman–Kittel–Kasuya–Yosida (RKKY)), as well as magnetic anisotropies, results in a very rich magnetic phenomenology^48^, including in some cases the presence of magnetic skyrmions (Fig. 3b). Skyrmions are topologically protected 3D spin textures which subtend the whole 4*π* steradians of the spin space with a resulting non-trivial integer topological charge. They may exist as truly localized solitary objects with continuous magnetization rotation. For the formation of skyrmion lattices, large IDMI values, and typically magnetic bias fields are needed. The magnitude of IDMI depends significantly on the electron orbit overlap, and thus on the film elements, their arrangement, temperature and strain. Except in some special cases^49^, IDMI is typically orders of magnitude lower than the symmetric exchange interactions, with its effective value decreasing with magnetic film thickness^50^. In films with smaller IDMI, this is nevertheless enough to break the inversion symmetry and select one of the two possible DW chiralities^51^, forming, for example, chiral bubbles (Fig. 3c).

Following the first theoretical predictions and experimental evidence of skyrmions in single-phase bulk magnetic materials with non-centrosymmetric crystallographic structures, research activities are now mostly focused on heterostructures with IDMI, due to their potential for applications^52^. Crucial aspects, such as creation, detection, manipulation and deletion of individual nanoscale skyrmions were first shown in Pd/Fe bilayers on Ir(111) utilizing spin-polarized scanning tunnelling microscopy^53^. The strong IDMI in single Fe monolayers on Ir(111) stabilizes a non-reconfigurable skyrmion lattice that exists for temperatures up to 30 K and magnetic fields up to 9 T. The stabilization of skyrmions at room temperature has been achieved by increasing the film thickness of the transition metal, providing interlayer exchange interaction, and engineering asymmetric multilayer stacks with distinct heavy-element films for additive IDMI at top and bottom interfaces of the magnetic layer^54^. The corresponding skyrmion core/bubble size at room temperature is on the order of 10–100 nm^54^,^55^, and hence 10–100 times larger than those in Fe/Ir(111).

Owing to their small size, highly non-collinear spin texture and topological protection, they can act as localized mobile data for spintronic applications. To write, read and delete these skyrmions/chiral bubbles, several mechanisms have been proposed and some demonstrated. These include spin-transfer/spin–orbit torque via spin-polarized current, magnons, spin Hall effect and electric field-driven magnetoelectric coupling^53^,^56^,^57^,^58^. The potentially low pinning and small scattering cross-section of skyrmions originating from their spatial confinement implies both small on-set current density and large lateral deflection due to an accompanying Magnus force^59^. Current-independent unidirectional motion along nanostrips may be enforced via strain, exchange or anisotropy modulations^58^, or eventually considering antiferromagnetic (AF) skyrmions^60^. In practice, pinning/scattering at grain boundaries caused by structural and magnetic imperfection in polycrystalline and amorphous films results in larger current densities required for coherent motion^54^.

### Vertical data motion using magnetic solitons

The extension of magnetic data mobility to the vertical direction has been recently realised by means of magnetic solitons in AF superlattices; this opens a new route to 3D magnetic random access memory devices with out-of-plane functionality. The term soliton refers here to a (topological) kink in a discrete chain of spins^61^, and is equivalent to an AF wall. The idea is analogous to the one employed in Nano-Magnetic Logic^62^, but instead of using soliton motion in dipolar-coupled nanomagnets positioned on the substrate plane, solitons are moved perpendicularly to the substrate plane in magnetic superlattices, where the coupling between magnetic thin films is provided by non-magnetic spacers via AF RKKY interactions. The system can be represented in its simple form by a 1D chain of spins (Fig. 3d), following a macrospin approximation, where layer thickness (*t*), anisotropy (*K*) and surface coupling (*J*) energies rule the physics of the system. Depending on the ratio between RKKY coupling and anisotropy fields (*H*_*J*_=|*J*|/(*μ*_0_*M*_s_*t*) and *H*_u_=2 *K*/(*μ*_0_*M*_s_) respectively) three regimes regarding magnetic texture and motion of solitons can be distinguished, as schematically shown in Fig. 3d.

For high RKKY couplings, solitons are wide, comprising a large number of layers, with those spins forming the soliton deviating a great amount from the anisotropy direction. In systems with very high coupling/anisotropy ratios, the rotation results in helical spin structures^63^. For soliton nucleation, the surface-spin flop transition can be exploited^64^, and reliable unidirectional propagation can be achieved by creating asymmetric superlattices, where thickness and anisotropy of a gate edge layer is tuned differently from the others. Following this approach, asynchronous motion under external magnetic fields using gates formed by single^65^ and highly coupled multiple layers^66^ has been achieved. As RKKY coupling decreases, becoming comparable to anisotropy, solitons get narrower, acquiring a well-defined magnetic moment. Interestingly, macrospin and micromagnetic simulations predict how in this case, external rotating magnetic fields can couple to the soliton magnetic configuration, resulting in synchronous propagation with the field, where the direction of motion is defined by the soliton chirality and sense of rotation of the field. This could be exploited to create bi-directional multi-bit vertical shift registers^67^.

Moreover, in spite of losing their chirality, synchronous motion is also possible for sharp solitons (when anisotropy becomes much larger than coupling). This has been realised under external oscillating magnetic fields, in Ising systems with strong perpendicular magnetic anisotropy, where the intrinsic layer coercivity (*H*_c_) substitutes *H*_u_ as relevant parameter for operation. By carefully engineering the thickness of the layers and the exchange coupling between them, it is possible to break inversion symmetry, which induces a unidirectional ratchet action^68^. Figure 3e shows schematically the structure of a ratchet superlattice, where layer thickness and interlayer RKKY coupling oscillate periodically between two possible values. Such a scheme makes possible the synchronous propagation of a soliton with perpendicular oscillating magnetic fields^68^. Remarkably, and following this approach, a unidirectional soliton shift register with vertical functionality equivalent to ∼20 transistors has been achieved within a thickness of 2 nm (ref. 68). Simple logic operations involving soliton–soliton annihilation have been realised as well^69^. Analogous ratchet schemes are possible; for example, a similar behaviour has been achieved by designing a superlattice with same thickness for all magnetic layers, and a combination of AF and ferromagnetic interlayer couplings^70^.

The aforementioned results refer to studies in extended films. Future studies are expected in laterally patterned nanostructures, to investigate the effect of rotating fields and dipolar interactions on the domain formation and soliton operation^71^,^72^. The use of different mechanisms for propagation, such as spin-transfer torque^73^, microwave excitations^74^ or all optical switching^75^, are also anticipated. Additionally, a new energy storage concept based on the continuous injection of solitons via the rotation of one edge spin in a cork-screw fashion, has been recently proposed^63^.

## Future applications of 3D nanomagnetism

### Sensing and actuation

The use of 3D nanomagnets for sensing and actuation applications has a huge potential in many scientific and technological areas. Some of the novel functionalities foreseen in the field of 3D magnetic nano-sensing are the substantial increase in surface-to-volume ratio with respect to planar systems, their capability to probe vectorial fields, an ad hoc geometrical design to match a sensed structure, and the exploitation of mechanical and thermal effects present in suspended structures which may couple to their magnetic response.

In particular, 3D magnetic nanomembranes (forming part of the large family of emerging flexible devices, for example, electronics displays and solar cells) which are transforming rigid electronics into light and shapeable elements, have already proven to bear great universal potential as flexible magnetic sensors. Remarkably, their magnetic sensitivity, harnessing giant magnetoresistance or giant magnetoimpedance, is similar (or in some cases more than one order of magnitude larger^76^) to devices produced on Si wafers, even after demanding manipulations^4^,^77^. This is due to the continuity of the magnetization along their closed surface, that is, due to their topology^78^. These features have stimulated desire for their application in industry as precise and flexible positioning control of magnetic bearings in electric motors^79^, and as printable sensing paste for simple but versatile magnetic switches (for example, electric postcards^80^). Further potential applications in life sciences and diagnostics include light, mobile and affordable devices with great spatial localization so as to facilitate magnetoencephalography^76^ for early stage disease detection.

Additionally, 3D nanomagnets could be incorporated as sensors in a new generation of scanning probe microscopy methods for advanced imaging and spectroscopy. In fact, high aspect-ratio NWs have already been employed as ultra-sharp MFM tips, improving magnetic resolution down to 10 nm^81^ and overcoming limitations of standard triangular tips in probing 3D nanostructures^82^. Furthermore, nano-spheres grown on top of cantilevers have been exploited to perform ferro-magnetic-resonant force microscopy, measuring magnetization dynamics with high spatial resolution^83^. The development of new tip designs incorporating new geometries and materials can exploit high-order resonant cantilever modes to measure field gradient components along several directions in space^84^. This and other approaches^85^ could make possible the realization of scanning probe vectorial microscopy: mapping the three components of stray fields emanating from a magnetic system at nanoscale resolution.

Moreover, 3D nanostructures often make very good actuators: their larger volume compared to planar 2D structures increases the torque and force generated by electromagnetic fields and strain. They are often free to deform reversibly, with interest for the NEMS industry, nanoelectronics, sensing, robotics and studies in the quantum regime^86^,^87^,^88^,^89^,^90^. In the case of 3D nanomagnets, additionally to standard electrical driving methods, remote magnetic actuation is possible via external magnetic fields^91^. This can be exploited to create nanomotors in fluids, where chiral helical structures^92^ or Janus particles^93^ have been controllably moved under rotating and oscillating magnetic fields, respectively. We foresee future works exploiting dipolar interactions, integrating different magnetic materials and more complex geometries, incorporating optical and electrical readout methods, and further integration into biological environments.

In particular, 3D nano- and micro-scale mechanical actuators are a key part of the tool-kit of mechano-biology^94^,^95^, an emerging field which tries to understand the role of cell and tissue mechanics in diseases. Single 3D magnetic nanostructures subjected to laboratory-scale magnetic fields can generate tens of nanonewton of force locally and controllably to cells. This is sufficient to probe the phenotype of cells^96^, mechanically induce differentiation of bone stem cells^97^, burst payload-carrying neural stem cells^98^ and destroy cancer cells^99^. Additionally, mammals use motile cilia (flagella-like microtubules) beating together to sweep objects through organs (such as removing dirt from the lungs or transporting eggs through female Fallopian tubes). Magnetic NWs could perhaps form artificial cilia^100^, transporting reagents through on-chip microfluidic channels.

### Towards data storage and the Internet-of-Things

3D magnetic nanostructures could form the basis of a new storage class of non-volatile memories, in which multiple data bits are stored magnetically above a single electronic memory cell on an integrated circuit. This is sustained by the impressive progress on ‘magnetic data mobility' in the last few years, in which physical effects such as spin-transfer torque^101^,^102^ allow digital information to be propagated through nanomagnets via magnetic entities such as DWs, skyrmions or solitons. The realization of 3D magnetic memories could be implemented using perhaps the original racetrack architecture^103^. Alternatively, existing magnetic random access memory technology could be enhanced by increasing the number of data storing magnetic layers in the stack, such that each cell can store a data word instead of a data bit^104^. In both of these cases, digital shift-register action is an essential ingredient^68^, allowing data bits in magnetic form to be sequentially pumped into the nanomagnet during writing and then pumped back out during reading. Also, a recent trend in microelectronics is to create 2.5-dimensional devices^105^ by stacking multiple thin substrates on top of each other in a single package, with wire interconnects running between layers. Magnetic NWs could use these ideas to enable 2.5-dimensional spintronic devices (that is, chips which use both electronic and magnetic components), allowing chip designers to connect electronic parts using electrical wires and magnetic parts using magnetic NWs.

Magnetic interconnect is a particularly interesting idea because it can host both magnetic DWs^106^ and magnetic spin waves^107^. The former would carry digital information in a very compact form but at low speed, while the latter would offer long-range transmission. Intriguingly, the magnetic hysteresis and non-linearity present in many magnetic NWs mean that the interconnect is potentially also both a memory element and a logic gate^108^, opening up new computing architectures in which the traditional boundaries between memory, logic and interconnect are eliminated. Such chips could even go beyond conventional Boolean logic and implement neuromorphic computing architectures^109^ in which 3D networks of magnetic NWs mimic the neurons and synapses in living brains.

However, significant challenges still remain before such applications become reality. In particular, modern microelectronics makes great use of precision interfaces between materials; many of the growth techniques used for 3D structures do not currently offer sufficient purity and control to engineer interfaces with single atomic layer precision. Also, while planar microchips successfully integrate and connect 10^8^ transistors on a single chip, complex interconnectivity in 3D is still to be developed. A human brain may connect up to 10^4^ synapses to each neuron^110^; there are currently no known 3D fabrication techniques able to achieve this.

**Figure 4:**
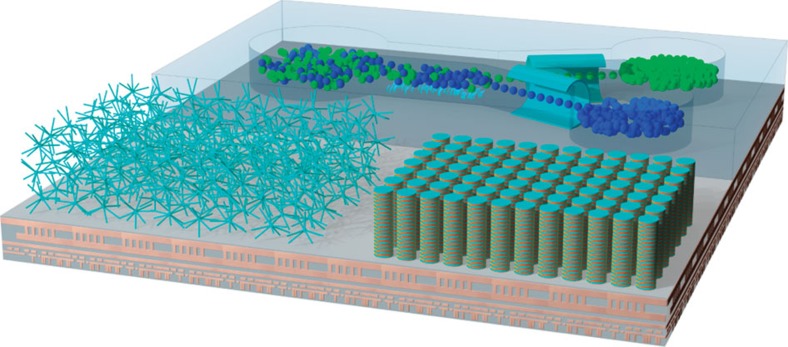
Futuristic vision of an Internet-of-Things chip integrating 3D magnetic nanostructures. The chip is comprised of a microfluidic channel with two types of magnetic nanoparticles flowing from a common reservoir. The motion of fluid is achieved by magnetic NWs acting as artificial cilia. After particle separation by chemical means, individual particles entering each channel are detected via a nanomembrane magnetic flexible sensor. An array of vertical soliton conduits acts as an ultra-high-density 3D storage device, with read/write operations taking place on the substrate. Neuromorphic computing processes are carried out by a dense array of interconnected NWs.

The integrated circuits of the future will increasingly incorporate a wider range of technologies onto a single die to form full systems (Fig. 4). The inclusion of physical sensors and energy harvesters into the menu of devices available to designers is particularly important for the Internet-of-Things. 3D magnetic nanostructures may find a role in both of these, where large surface areas, the ability to easily strain, and the possibility of sustaining temperature differentials are often important and difficult to achieve in 2D planar geometries. Recent advances in spin caloritronics^111^ and multiferroic materials^112^ will provide the necessary interconversion between the electronic, magnetic and thermal worlds.

## Perspectives

The move from 2D to 3D is not only a current trend within nanomagnetism, but is part of a wider theme within nanotechnology in general. Nanoelectronics, nanophotonics, energy storage and harvesting, and nanomedicine, all stand to benefit from a new era of greener, more capable, multi-functional technologies brought about by moving into the third dimension. Despite the great challenges ahead, recent advances in bottom-up lithography, microscopy and computational techniques make its future realization feasible.

3D nanomagnetism, still in its infancy, needs to fulfil key milestones in the future. The experimental realization of spintronic devices requires high-quality materials, for example, DW motion may be severely affected by the presence of defects. Achieving such a quality with unconventional synthesis methods is still work in progress. Also, next-generation 3D nanomagnets will require advanced magnetic materials such as Heusler alloys, multi-layered and epitaxial thin films, and highly functional interfaces. These barriers could be overcome using fabrication strategies which combine multiple synthesis techniques, including innovative ways to exploit differential strain^113^. The low throughput currently achievable in 3D nano-printing could be significantly enhanced by moving from the gas phase to the liquid phase^114^. This may enable the development of complex architectures, mass fabrication and the creation of multi-functional designs. Future years will also see rapid progress in the synthesis of novel magnetic materials such as 3D nanocomposites^115^, where strong electric fields at interfaces or magnetostrictive effects could be used for energy harvesting. Magnetic tools able to probe 3D nanomagnets are essential as well. Vectorial magnetometry and magnetic microscopy of nanostructures require a phenomenal combined effort comprising instrumentation, electronics and data analysis. Recent progress in magnetic microscopy methods^116^,^117^,^118^,^119^, pushing further spatial and temporal resolution, is expected to bring new opportunities in this area. An essential cornerstone still missing due to its technical complexity is the development of methodologies^120^ to electrically contact individual 3D nano-geometries.

Advances in these areas are essential to study 3D spin textures further. Recent experimental work has confirmed theoretical and computational predictions about their magnetic structure and static behaviour. The next step is now to focus on the experimental realization of specific dynamic features driven by different mechanisms. These are often related to the interplay between the chirality and/or curvature of the boundaries of the texture itself and the chiral nature of the Landau–Lifshitz equation. Interest is also growing in composite spin textures such as coupled bilayers^121^, where flux closure and moment compensation can enhance magnetic DW motion within the plane, as well as leading to motion between horizontal planes. The search for and realization of ever more complex geometries will continue, for example, by connecting planar and vertical structures, creating core-shell 3D objects, or exploiting topological effects via the interplay between 3D shapes and spin configurations^122^. The possibility of creating complex 3D networks of nanomagnets could lead to new computational paradigms^123^, and is also expected to elucidate open questions regarding effects involving numerous interacting magnetic particles, for example, artificial spin-ice lattices^124^. Three-dimensional nanomagnetism, with its vast amount of unexplored science and huge potential to impact society, is a fascinating new research area, which will flourish in the years to come.

## Additional information

**How to cite this article:** Fernández-Pacheco, A. *et al*. Three-dimensional nanomagnetism. *Nat. Commun.* **8,** 15756 doi: 10.1038/ncomms15756 (2017).

**Publisher's note:** Springer Nature remains neutral with regard to jurisdictional claims in published maps and institutional affiliations.

## Figures and Tables

**Figure 1:**
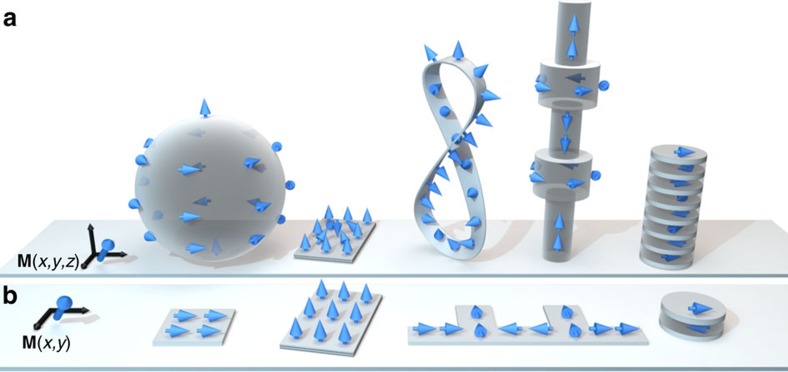
Towards three-dimensional nanomagnetism. Schematic view comparing some examples of geometries and magnetic configurations (indicated by blue arrows) for (**a**) 3D and (**b**) 2D nanomagnetism. The dependence of the magnetization **M** on spatial coordinates (black arrows) is indicated for both cases. New synthesis, characterization and computational methods have the potential to make the leap to 3D. The combination of more complex magnetic states and additional degrees of freedom in 3D nanomagnets leads to the emergence of new physical phenomena, which may find applications in multiple areas. (**a**) Examples of 3D nanomagnets, from left to right: magnetic sphere with vortex configuration. Magnetic thin film element with a skyrmion. Symmetry breaking is caused by bulk or interfacial Dzyaloshinskii-Moriya interaction. Möbius strip with perpendicular magnetization; a DW is present in the ground state due to the object's topology. Cylindrical NW with modulated diameter, with different magnetic configurations depending on the diameter. Antiferromagnetic (AF) superlattice (interlayers not shown for clarity) with a wide soliton in the middle. (**b**) Examples of 2D nanomagnets, from left to right: Single-domain magnet. Magnetic multi-layered element with perpendicular anisotropy. Nanostrip with protrusions for DW trapping. Bi-layered magnet with AF coupling due to indirect exchange via an interlayer (not shown for clarity).

## SYNTHESIS.

2D nanomagnetism has been mostly developed via the combination of physical vapour deposition (PVD) and top–down lithography. Progress in 3D nanomagnetism relies however on new synthesis methods and strategies. The three main routes currently used for this purpose are physical, chemical and 3D nano-printing methods (see figure).

PVD can be extended to 3D if the deposition is done onto previously patterned 3D scaffolds, instead of onto flat substrates. Such scaffolds include those created by two-photon direct-write optical lithography^125^,^126^ and self-assembled methods^127^,^128^. However, this method faces challenges concerning conformal deposition and material shadowing. The anisotropic character of PVD has nevertheless been exploited to create ultra-small nano-helices^129^,^130^ presenting a strong magneto-chiral dichroic effect^131^. A complementary approach for 3D manufacturing based on thin films is rolled-up nanotechnology^132^. Here, differential strain acting on films provides a means to engineer nanomembranes of virtually any material^31^.

Chemical synthesis techniques are generally more suited to deliver 3D nanostructures. Using heterogeneous nucleation methods, assemblies of rods, dumbbells or branched networks can be produced^133^, with potential applications as rare-earth-free magnets^134^ and magnetic recording media^135^. However, the most common approach uses 3D-templates, exploiting the isotropic character and conformal growth achieved in electroplating, electroless plating and atomic layer deposition. Anodised alumina matrices are the most common templates, producing arrays of high vertical aspect-ratio NWs^136^, NTs^137^, core-shell^138^ and multi-layered NWs^139^, formed by a wide variety of materials. The remarkable control achieved over interfaces between materials during co-deposition has made possible spin-transport effect studies^140^,^141^. However, atomic-scale control over the thickness is not possible yet, hindering the synthesis of perpendicular materials based on surface anisotropy. Truly 3D template manufacturing is becoming available thanks to new methods to modulate template diameters along the pore length^142^,^143^, and latest advances in block-copolymer^144^ and other self-assembly methods. Complex 3D geometrical textures such as inverse opal lattices^145^ and gyroids^146^ have been realised, for applications in magnetic collective phenomena studies, for example, 3D spin ice.

The third route comprises of 3D nano-printing via focused electron beam induced deposition (FEBID)^147^. FEBID is an emerging direct-write patterning technique able to prototype individual complex 3D systems with resolution of a few tens of nanometre, in times ranging from seconds to a few minutes. Unlike in the past, FEBID is not restricted to the growth of carbonaceous materials, but polycrystalline metallic cobalt and iron can be synthesized^148^,^149^. Progress towards the growth of magnetic alloys^150^,^151^ and functional spintronic interfaces^152^ have been recently reported. 3D geometries grown by FEBID include vertical NWs for MFM^153^, spintronic applications^82^,^154^ and 3D actuators^91^, as well as nano-spheres^83^, nano-helices^154^, nano-ellipsoids^155^ and nano-bridges^156^. Future works should scale the growth to larger areas^157^, and will exploit new purification stategies^158^,^159^,^160^, as well as recently developed simulation growth algorithms combining continuum models and Monte Carlo methods^161^.

**Three main routes for synthesizing 3D nanomagnets**. (**a**) A buckyball made by two-photon optical lithography, with cobalt sputtered on top. Reprinted with permission from ref. 125. Copyright (2015) by the American Physical Society. (**b**) Cylindrical NWs electrodeposited from a CoSO_4_ electrolyte on alumina templates (see inset). Reproduced from ref. 136 with the permission of AIP Publishing. (**c**) A nano-spiral created by 3D nano-printing using Co_2_(CO)_8_. Adapted from ref. 154, Nature Publishing Group.

## MAGNETIC CHARACTERIZATION.

Magnetic characterization techniques at the nanoscale have been mostly developed for and are suitable to probe planar systems. Their extension to the study of 3D nanomagnets, presenting complex vectorial spin textures and geometries is far from trivial, and requires new methodologies and multi-technique approaches. In particular, magnetometry techniques able to probe single planar nanostructures^162^,^163^,^164^ has been an essential factor for the progress of nanomagnetism in recent years. These studies have been extended to 3D for the simplest geometries, such as NWs and NTs, following an intermediate micromanipulation step, which places them flat at a particular position on a substrate. Using this approach, the switching field angular dependence of cylindrical NWs has been measured, to determine the mechanism behind magnetization reversal. This has been done by detecting the emanating magnetic flux using nano-SQUID^162^ and their magneto-optical response using Kerr effect^154^. Analogously, dynamic-cantilever magnetometry has been employed in NWs and NTs glued at the edge of a cantilever, detecting the frequency shift of oscillation caused by the magnetic torque under external fields^165^,^166^. Switching probability measurements also allow a method to probe the thermally activated nature of the switching process^162^,^167^. For the advance of the area, new magnetometry methods for measurements of as grown 3D suspended nanostructures, which do not require such complicated micromanipulation intermediate steps are desired. This has been recently reported using magneto-optical Kerr effect^154^, where the switching fields of 100 nm-diameter, several μm-long cobalt NWs grown tilted with respect to the substrate were measured. The NW angle creates a projection on the substrate of the order of the laser diameter (5 μm), making the magneto-optical signal measurable.

A traditional way to probe magnetism in relatively large volumes consists of using neutron scattering techniques^168^. For instance, polarized neutron reflectivity has been used to determine the magnetic state of superlattices during soliton propagation^64^,^66^, and small-angle scattering has revealed the formation of skyrmion lattices in non-centrosymmetric bulk crystals^169^,^170^. Recently developed neutron tomography methods^171^ enable the vectorial reconstruction of magnetic domains with resolution of ∼100 μm. In the case of spatially localized 3D objects, magnetic arrays such as wires and nanoparticles can be investigated in a small-angle scattering geometry^145^. However, the analysis soon reaches its limits when many parameters contribute to the recorded signal, such as distributions, imperfect texturing or correlations. A powerful approach consists of probing a highly ordered array of textured objects, where the scattering pattern can be analysed in a similar way as crystallography makes use of the reciprocal space, to extract detailed information of the structure of the lattice cell^172^.

**Magnetometry measurements on 3D cylindrical NWs**. In (**a**), the wire is placed on top of a nano-SQUID using a micromanipulator. Scale bar, 1 μm. Reprinted with permission from ref. 162. Copyright (1996) by the American Physical Society. In (**b**), the magnetic switching of free-standing NWs forming 45^o^ with the substrate plane is detected using spatially resolved magneto-optical Kerr effect. Additional sources of noise result from the mechanical motion via vibrations and coupling with the external field, and the large amount of diffusive scattering produced by a non-planar surface. Figure taken from ref. 154, Nature Publishing Group.

## MAGNETIC MICROSCOPY IN 3D.

Imaging 3D magnetic textures requires techniques with nanoscale spatial resolution, quantitative information and sensitivity to the vector character of the spin. Transmission electron microscopies (TEM) and X-ray microscopies (XM) are currently the techniques most suitable for these studies^173^. Magnetic lateral spatial resolution ranges from tens of nm for XM, down to sub-10 nm for TEM. Recent advances in Lorentz lens aberration corrections have enabled magnetic imaging at zero field down to 0.5 nm (ref. 174). XM has some other advantages, such as the straightforward application of electromagnetic fields, and the possibility to perform time-resolved experiments exploiting the temporal structure of pulsed X-ray sources.

TEM and XM are sensitive to either the projection of the magnetic induction vector **B** onto the plane perpendicular to the electron path (TEM), or to the component of the magnetization **M** along the photon direction (XM). The standard methodology to investigate 3D magnetic textures thus consists of comparing the information of a 2D projection for a given angle, with the one obtained by micromagnetic simulations. For instance, the magnetic configuration of a skyrmion lattice in FeCoSi and FeGe thin films has been determined using Lorentz TEM^175^,^176^. Using off-axis Electron Holography, multi-layered NWs reveal different magnetic configurations depending on their microstructure and composition^174^. Also, remanent states of Ni cylindrical NWs^23^ and multi-layered Co/Ni NWs^177^ give experimental evidence of some of the types of DWs discussed in the main text.

The increasing demand on 3D imaging techniques is leading to novel concepts that go beyond the aforementioned approach, by extending nano-tomography of scalar quantities^178^ to magnetic investigations. By rotating the stage along an axis and acquiring 2D projections of **B** at several angles, both chirality and core orientation of vortices in magnetic disks have been determined^179^, and the magnetic structure of NWs and nano-spirals has been imaged^180^,^181^,^182^. Whereas in special symmetries full vector-field tomographic reconstructions of **B** is feasible by one simple projection^183^, in general projection series need to be recorded while rotating the sample about at least two axes. This has been applied to reconstruct the vectorial magnetic configuration of Permalloy squares^184^ and of magnetic vortices in two stacked disks^185^.

In the realm of XMs, new 3D imaging methodologies have been reported so far for transmission-X-ray photoemission electron microscopy (XPEEM)^128^,^186^, ptychography^125^ and magnetic X-ray tomography (MXT)^187^. In XPEEM, the 3D magnetic configuration of core-shell NWs has been visualized, by collecting both secondary photoelectrons emanating from the NW surface (conventional XPEEM) and from the substrate (XMCD shadow contrast)^186^. This same method has been employed to prove the existence of Bloch point DWs^24^ and to image magnetization reversal processes in buried layers of curved magnetic nanomembranes^188^. First demonstrations of MXT vectorial tomography have been recently obtained for 3D nanomembranes^187^ and films with canted magnetization^189^.

**Magnetic vectorial tomography using electron and X-ray methods**. (**a**) Reconstructed 3D magnetic vectorial potential (red arrows) of a Permalloy square using Lorentz electron microscopy. Reprinted with permission from ref. 184. Copyright (2010) by the American Physical Society. (**b**) Perspective SEM view of a standing rolled-up nickel nanomembrane overlaid with XMCD shadow contrasts recorded at various projection angles from the planar substrate using XPEEM. Scale bar, 5 μm. Adapted from ref. 187, Nature Publishing Group.

## COMPUTATIONAL METHODS.

Micromagnetic simulations, rooted in a rigorous implementation of the fundamental equations of the theory of nanomagnetism, describe the spatial and temporal evolution of the continuous vector field of the magnetization and of the magnetostatic field, and can incorporate spin-torque effects. This powerful computational framework has provided a deep understanding of experimental observations and promoted research toward new effects^190^.

The experimental realization of 3D magnetic nanostructures necessitates micromagnetic investigations at a large scale. Progress in computer hardware and code development has made it possible to simulate micron-sized samples with an ordinary desktop computer. Owing to enhanced numerical methods and massively parallel computations, the upper limit in size is no longer a critical issue. Similarly, memory limits, which used to be hit easily several years ago, no longer represent a serious concern. Today, micromagnetic modelling requires high-performance computing for other reasons, such as the simulation of large arrays of interacting nanomagnets or hybrid atomistic-continuum simulations. Programmable Graphical Processing Units (GPUs) represent an impressively powerful approach to high-performance computing for these purposes^191^,^192^,^193^. In the past years, GPU-based personal supercomputing has outpaced the use of traditional large-scale supercomputing facilities, since they are inexpensive and require much less energy. As a result, post-processing, visualization and storage of huge amounts of simulated data have emerged as new challenges.

Another important point is which of the two possible branches, either finite difference methods (FDM) or finite element methods (FEM), are employed. FEM can model arbitrary shapes with high accuracy, making it more suitable for simulating complex 3D shapes, especially if curved surfaces are involved. On the other hand, FDM generally has higher speed for calculating magnetization dynamics and is easier to use and implement. In the past, the regular grid required by FDM (allowing for the use of Fast-Fourier Transform methods for the calculation of magnetostatic fields) allowed for shorter computational times and lower memory requirements. However, current matrix compression schemes employed now by FEM have largely removed these differences. Still, owing to the regular discretization grid of the FDM, the time integration of the Landau–Lifshitz–Gilbert is generally less critical than in FEM, which allows for larger time steps and thus faster integration.

Although micromagnetic simulations studies of 3D structures have proven to be generally very reliable, the continuum theory behind these algorithms ceases to be valid in certain cases^194^. Strongly inhomogeneous magnetic structures^195^, laser-induced magnetization dynamics, and sample sizes approaching the atomic limit call for extensions to the standard approach. In such cases, atomistic magnetic models^196^,^197^ can be introduced. These models may either replace a micromagnetic code, or complement it in the form of a hybrid model (see figure) where atomistic magnetic moments are coupled to a surrounding continuum^39^.

**Multiscale micromagnetic and atomistic models**. The magnetic sphere combines both models to simulate the structure and dynamics of Bloch points. The central blue and outer orange areas correspond to the space where either a purely atomistic or a purely micromagnetic model is used to calculate the exchange interaction between spins, with the green area in between as the interface between both. Reprinted with permission from ref. 39. Copyright (2014) by the American Physical Society.

## References

[b1] Editorial. Memory with a spin. Nat. Nanotechnol. 10, 185–185 (2015). 25740125 10.1038/nnano.2015.50

[b2] Hoffmann A. & Bader S. D. Opportunities at the frontiers of spintronics . Phys. Rev. Appl. 4, 047001 (2015).

[b3] McCray W. P. How spintronics went from the lab to the iPod. Nat. Nanotechnol. 4, 2–4 (2009). 19119267 10.1038/nnano.2008.380

[b4] Melzer M. . Imperceptible magnetoelectronics. Nat. Commun. 6, 6080 (2015). 25607534 10.1038/ncomms7080 PMC4354162

[b5] Makarov D., Melzer M., Karnaushenko D. & Schmidt O. G. Shapeable magnetoelectronics. Appl. Phys. Rev. 3, 011101 (2016).

[b6] Koh I. & Josephson L. Magnetic nanoparticle sensors. Sensors 9, 8130–8145 (2009). 22408498 10.3390/s91008130 PMC3292100

[b7] Chen R., Romero G., Christiansen M. G., Mohr A. & Anikeeva P. Wireless magnetothermal deep brain stimulation. Science 347, 1477–1480 (2015). 25765068 10.1126/science.1261821

[b8] Faure B. . 2D to 3D crossover of the magnetic properties in ordered arrays of iron oxide nanocrystals. Nanoscale 5, 953–960 (2013). 23238262 10.1039/c2nr33013j

[b9] Sun H. . Self-organized honeycomb structures of Mn _12_ single-molecule magnets. J. Phys. Chem. B 113, 14674–14680 (2009). 19824615 10.1021/jp906520j

[b10] Stamps R. L. . The 2014 magnetism roadmap. J. Phys. D Appl. Phys. 47, 333001 (2014).

[b11] Waldrop M. M. The chips are down for Moore's law. Nature 530, 144–147 (2016). 26863965 10.1038/530144a

[b12] Parkin S. & Yang S.-H. Memory on the racetrack. Nat. Nanotechnol. 10, 195–198 (2015). 25740128 10.1038/nnano.2015.41

[b13] Mattheis R., Glathe S., Diegel M. & Hübner U. Concepts and steps for the realization of a new domain wall based giant magnetoresistance nanowire device: From the available 24 multiturn counter to a 212 turn counter. J. Appl. Phys. 111, 113920 (2012).

[b14] Hrkac G., Dean J. & Allwood D. A. Nanowire spintronics for storage class memories and logic. Philos. Trans. A Math. Phys. Eng. Sci. 369, 3214–3228 (2011). 21727122 10.1098/rsta.2011.0138

[b15] Jamet S., Rougemaille N., Toussaint J. C. & Fruchart O. in Magnetic nano- and microwires: design, synthesis, properties and applications (ed. Vazquez M. 783–811Woodhead (2015).

[b16] McMichael R. D., Eicke J., Donahue M. J. & Porter D. G. Domain wall traps for low-field switching of submicron elements. J. Appl. Phys. 87, 7058–7060 (2000).

[b17] Arrott A., Heinrich B. & Aharoni A. Point singularities and magnetization reversal in ideally soft ferromagnetic cylinders. IEEE Trans. Magn. 15, 1228–1235 (1979).

[b18] Feldtkeller E. Mikromagnetisch stetige und unstetige Magnetisierungskonfigurationen. Z. Angew. Phys. 19, 530–536 (1965).

[b19] Malozemoff A. P., Slonczewski J. C. & Raymond W. Magnetic Domain Walls In Bubble Materials Academic Press (1979).

[b20] Thiaville A. & Nakatani Y. in Spin Dynamics in Confined Magnetic Structures III eds Hillebrands B., Thiaville A. 161–205Springer (2006).

[b21] Forster H. . Domain wall motion in nanowires using moving grids (invited). J. Appl. Phys. 91, 6914–6919 (2002).

[b22] Hertel R. Computational micromagnetism of magnetization processes in nickel nanowires. J. Magn. Magn. Mater. 249, 251–256 (2002).

[b23] Biziere N. . Imaging the fine structure of a magnetic domain wall in a Ni nanocylinder. Nano Lett. 13, 2053–2057 (2013) ***This paper demonstrates imaging of the internal structure of domain walls in cylindrical nanowires***. 23586647 10.1021/nl400317j PMC3650658

[b24] Da Col S. . Observation of Bloch-point domain walls in cylindrical magnetic nanowires. Phys. Rev. B 89, 180405 (2014) ***This article reports experimental proof of the existence of Bloch-point domain walls in cylindrical nanowires***.

[b25] Landeros P. . Reversal modes in magnetic nanotubes. Appl. Phys. Lett. 90, 102501 (2007).

[b26] Hertel R. Curvature-induced magnetochirality. SPIN 3, 1340009 (2013) ***This work presents a theoretical discussion of magnetochiral effects induced by surface curvature in ferromagnetic systems***.

[b27] Gaididei Y., Kravchuk V. P. & Sheka D. D. Curvature effects in thin magnetic shells. Phys. Rev. Lett. 112, 257203 (2014). 25014827 10.1103/PhysRevLett.112.257203

[b28] Sheka D. D. . Curvature effects in statics and dynamics of low dimensional magnets. J. Phys. A Math. Theor. 48, 125202 (2015).

[b29] Landeros P. & Nüńez A. S. Domain wall motion on magnetic nanotubes. J. Appl. Phys. 108, 033917 (2010).

[b30] González A. L., Landeros P. & Núñez Á. S. Spin wave spectrum of magnetic nanotubes. J. Magn. Magn. Mater. 322, 530–535 (2010).

[b31] Streubel R. . Magnetism in curved geometries. J. Phys. D Appl. Phys. 49, 363001 (2016) ***A comprehensive topical review of 3D curved magnetic geometries***.

[b32] Ryu K.-S., Thomas L., Yang S.-H. & Parkin S. Chiral spin torque at magnetic domain walls. Nat. Nanotechnol. 8, 527–533 (2013) ***This article provides an elucidation of the mechanisms behind chiral spin torques in nanostrips made of ultra-thin films***. 23770808 10.1038/nnano.2013.102

[b33] Emori S., Bauer U., Ahn S.-M., Martinez E. & Beach G. S. D. Current-driven dynamics of chiral ferromagnetic domain walls. Nat. Mater. 12, 611–616 (2013) ***This article demonstrates chiral spin torque in Neel-type domain walls in nanostrips***. 23770726 10.1038/nmat3675

[b34] Miron I. M. . Fast current-induced domain-wall motion controlled by the Rashba effect. Nat. Mater. 10, 419–423 (2011). 21572411 10.1038/nmat3020

[b35] Schryer N. L. & Walker L. R. The motion of 180° domain walls in uniform dc magnetic fields. J. Appl. Phys. 45, 5406–5421 (1974).

[b36] Yan M., Andreas C., Kákay A., Garcïa-Sänchez F. & Hertel R. Fast domain wall dynamics in magnetic nanotubes: suppression of Walker breakdown and Cherenkov-like spin wave emission. Appl. Phys. Lett. 99, 122505 (2011) ***This numerical study predicts the Spin-Cherenkov effect in nanotubes***.

[b37] Yan M., Andreas C., Käkay A., Garcïa-Sänchez F. & Hertel R. Chiral symmetry breaking and pair-creation mediated Walker breakdown in magnetic nanotubes. Appl. Phys. Lett. 100, 252401 (2012).

[b38] Hertel R. Ultrafast domain wall dynamics in magnetic nanotubes and nanowires. J. Phys. Condens. Matter 28, 483002 (2016). 27701143 10.1088/0953-8984/28/48/483002

[b39] Andreas C., Kákay A. & Hertel R. Multiscale and multimodel simulation of Bloch-point dynamics. Phys. Rev. B 89, 134403 (2014) ***An application of multiscale modelling for the study of 3D spin textures***.

[b40] Hertel R. & Andreas C. in Magnetic Nano- and Microwires. Design, Synthesis, Properties and Applications ed. Vazquez M. 653–677Woodhead (2015).

[b41] Shinjo T., Okuno T., Hassdorf R., Shigeto K. & Ono T. Magnetic vortex core observation in circular dots of permalloy. Science 289, 930–932 (2000) ***This paper describes the experimental observation of vortices in dots***. 10937991 10.1126/science.289.5481.930

[b42] Wachowiak A. . Direct observation of internal spin structure of magnetic vortex cores. Science 298, 577–580 (2002). 12386329 10.1126/science.1075302

[b43] Burgess J. A. J. . Quantitative magneto-mechanical detection and control of the barkhausen effect. Science 339, 1051–1054 (2013). 23328394 10.1126/science.1231390

[b44] Streubel R., Fischer P., Kopte M., Schmidt O. G. & Makarov D. Magnetization dynamics of imprinted non-collinear spin textures. Appl. Phys. Lett. 107, 112406 (2015).

[b45] Zhu J.-G., Zheng Y. & Prinz G. A. Ultrahigh density vertical magnetoresistive random access memory (invited). J. Appl. Phys. 87, 6668 (2000).

[b46] Pylypovskyi O. V. . Coupling of chiralities in spin and physical spaces: the möbius ring as a case study. Phys. Rev. Lett. 114, 197204 (2015). 26024195 10.1103/PhysRevLett.114.197204

[b47] Zhang S., Levy P. & Fert A. Mechanisms of spin-polarized current-driven magnetization switching. Phys. Rev. Lett. 88, 236601 (2002). 12059385 10.1103/PhysRevLett.88.236601

[b48] Bode M. . Chiral magnetic order at surfaces driven by inversion asymmetry. Nature 447, 190–193 (2007). 17495922 10.1038/nature05802

[b49] Dupé B. . Tailoring magnetic skyrmions in ultra-thin transition metal films. Nat. Commun. 5, 101–103 (2014). 10.1038/ncomms5030 24893652

[b50] Nembach H. T., Shaw J. M., Weiler M., Jué E. & Silva T. J. Linear relation between Heisenberg exchange and interfacial Dzyaloshinskii–Moriya interaction in metal films. Nat. Phys. 11, 825–829 (2015).

[b51] Chen G. . Tailoring the chirality of magnetic domain walls by interface engineering. Nat. Commun. 4, 2671 (2013). 24154595 10.1038/ncomms3671

[b52] Wiesendanger R. . Nanoscale magnetic skyrmions in metallic films and multilayers: a new twist for spintronics. Nat. Rev. Mater. 1, 16044 (2016).

[b53] Romming N. . Writing and deleting single magnetic skyrmions. Science 341, 636–639 (2013). 23929977 10.1126/science.1240573

[b54] Woo S. . Observation of room-temperature magnetic skyrmions and their current-driven dynamics in ultrathin metallic ferromagnets. Nat. Mater. 15, 501–506 (2016) ***This work reports the creation of skyrmions at room temperature, driven by current pulses in magnetic racetracks***. 26928640 10.1038/nmat4593

[b55] Jiang W. . Blowing magnetic skyrmion bubbles. Science 349, 283–286 (2015) ***This article demonstrates injection and motion of skyrmionic bubbles at room temperature***. 26067256 10.1126/science.aaa1442

[b56] Hanneken C. . Electrical detection of magnetic skyrmions by tunnelling non-collinear magnetoresistance. Nat. Nanotechnol. 10, 1039–1042 (2015). 26436563 10.1038/nnano.2015.218

[b57] Seki S., Yu X. Z., Ishiwata S. & Tokura Y. Observation of skyrmions in a multiferroic material. Science 336, 198–201 (2012). 22499941 10.1126/science.1214143

[b58] Hsu P.-J. . Electric field driven switching of individual magnetic skyrmions. Nat. Nanotechnol. 12, 123–126 (2017). 27819694 10.1038/nnano.2016.234

[b59] Reichhardt C. & Reichhardt C. J. O. Noise fluctuations and drive dependence of the skyrmion Hall effect in disordered systems. New J. Phys. 18, 095005 (2016).

[b60] Barker J. & Tretiakov O. A. Static and dynamical properties of antiferromagnetic skyrmions in the presence of applied current and temperature. Phys. Rev. Lett. 116, 147203 (2016). 27104724 10.1103/PhysRevLett.116.147203

[b61] Cowburn R. P. Room temperature magnetic quantum cellular automata. Science 287, 1466–1468 (2000) ***This paper shows transport of magnetic information in isolated dots for nanomagnetic logic applications***. 10688790 10.1126/science.287.5457.1466

[b62] Niemier M. T. . Nanomagnet logic: progress toward system-level integration. J. Phys. Condens. Matter 23, 493202 (2011). 22121192 10.1088/0953-8984/23/49/493202

[b63] Vedmedenko E. Y. & Altwein D. Topologically protected magnetic helix for all-spin-based applications. Phys. Rev. Lett. 112, 017206 (2014). 24483928 10.1103/PhysRevLett.112.017206

[b64] te Velthuis S., Jiang J., Bader S. & Felcher G. Spin flop transition in a finite antiferromagnetic superlattice: evolution of the magnetic structure. Phys. Rev. Lett. 89, 127203 (2002). 12225121 10.1103/PhysRevLett.89.127203

[b65] Fernández-Pacheco A. . Controllable nucleation and propagation of topological magnetic solitons in CoFeB/Ru ferrimagnetic superlattices. Phys. Rev. B Condens. Matter Mater. Phys. 86, 104422 (2012).

[b66] Fernández-Pacheco A. . Magnetic state of multilayered synthetic antiferromagnets during soliton nucleation and propagation for vertical data transfer. Adv. Mater. Interfaces 3, 1600097 (2016).

[b67] Petit D., Mansell R., Fernández-Pacheco A., Lee J. H. & Cowburn R. P. in VLSI: Circuits for Emerging Applications ed. Wojcicki T. Ch. 12, CRC Press (2014).

[b68] Lavrijsen R. . Magnetic ratchet for three-dimensional spintronic memory and logic. Nature 493, 647–650 (2013) ***This article presents the development of soliton-based vertical shift registers***. 23364743 10.1038/nature11733

[b69] Lavrijsen R. . Multi-bit operations in vertical spintronic shift registers. Nanotechnology 25, 105201 (2014). 24531860 10.1088/0957-4484/25/10/105201

[b70] Mansell R. . A robust soliton ratchet using combined antiferromagnetic and ferromagnetic interlayer couplings. Appl. Phys. Lett. 106, 092404 (2015).

[b71] Lee J. H. . Soliton propagation in micron-sized magnetic ratchet elements. Appl. Phys. Lett. 104, 232404 (2014).

[b72] Lee J.-H. . Domain imaging during soliton propagation in a 3D magnetic ratchet. SPIN 3, 1340013 (2013).

[b73] Kudo K. . Resonant magnetization switching induced by spin-torque-driven oscillations and its use in three-dimensional magnetic storage applications. Appl. Phys. Express 8, 103001 (2015).

[b74] Suto H. . Three-dimensional magnetic recording using ferromagnetic resonance. Jpn J. Appl. Phys. 55, 07MA01 (2016).

[b75] Lambert C.-H. . All-optical control of ferromagnetic thin films and nanostructures. Science 345, 1337–1340 (2014). 25147280 10.1126/science.1253493

[b76] Karnaushenko D. . Self-assembled on-chip-integrated giant magneto-impedance sensorics. Adv. Mater. 27, 6582–6589 (2015). 26398863 10.1002/adma.201503127

[b77] Melzer M. . Stretchable magnetoelectronics. Nano Lett. 11, 2522–2526 (2011). 21557570 10.1021/nl201108b

[b78] Makarov D., Ortix C. & Baraba L. Functional magnetic nanomembranes. SPIN 3, 1302001 (2013).

[b79] Ernst D. . Packaging technologies for (Ultra-)thin sensor applications in active magnetic bearings. in Proceedings of the 2014 37th International Spring Seminar on Electronics Technology 125–129IEEE (2014).

[b80] Karnaushenko D., Makarov D., Yan C., Streubel R. & Schmidt O. G. Printable giant magnetoresistive devices. Adv. Mater. 24, 4518–4522 (2012). 22761017 10.1002/adma.201201190

[b81] Belova L. M., Hellwig O., Dobisz E. & Dan Dahlberg E. Rapid preparation of electron beam induced deposition Co magnetic force microscopy tips with 10 nm spatial resolution. Rev. Sci. Instrum. 83, 093711 (2012). 23020387 10.1063/1.4752225

[b82] Gavagnin M. . Free-standing magnetic nanopillars for 3D nanomagnet logic. ACS Appl. Mater. Interfaces 6, 20254–20260 (2014). 25296008 10.1021/am505785t PMC4251043

[b83] Guo F., Belova L. M. & McMichael R. D. Spectroscopy and imaging of edge modes in permalloy nanodisks. Phys. Rev. Lett. 110, 017601 (2013). 23383836 10.1103/PhysRevLett.110.017601

[b84] Mühl T. . Magnetic force microscopy sensors providing in-plane and perpendicular sensitivity. Appl. Phys. Lett. 101, 112401 (2012).

[b85] Schuhl A., Gaudin G., Sabon P., Zermatten P.-J. & Montaigne F. Integrated magnetometer and its manufacturing process. World Patent Organization patent WO/2011/092406 (2011).

[b86] Blick R. H., Qin H., Kim H.-S. & Marsland R. A nanomechanical computer—exploring new avenues of computing. New J. Phys. 9, 241–241 (2007).

[b87] Chaste J. . A nanomechanical mass sensor with yoctogram resolution. Nat. Nanotechnol. 7, 301–304 (2012). 22466856 10.1038/nnano.2012.42

[b88] Hanay M. S. . Inertial imaging with nanomechanical systems. Nat. Nanotechnol. 10, 339–344 (2015). 25822931 10.1038/nnano.2015.32 PMC5283574

[b89] Hanay M. S. . Single-protein nanomechanical mass spectrometry in real time. Nat. Nanotechnol. 7, 602–608 (2012). 22922541 10.1038/nnano.2012.119 PMC3435450

[b90] Aspelmeyer M., Kippenberg T. J. & Marquardt F. Cavity optomechanics. Rev. Mod. Phys. 86, 1391–1452 (2014).

[b91] Vavassori P., Pancaldi M., Perez-Roldan M. J., Chuvilin A. & Berger A. Remote magnetomechanical nanoactuation. Small 12, 1013–1023 (2016). 26766300 10.1002/smll.201503351

[b92] Walker D., Kübler M., Morozov K. I., Fischer P. & Leshansky A. M. Optimal length of low reynolds number nanopropellers. Nano Lett. 15, 4412–4416 (2015). 26030270 10.1021/acs.nanolett.5b01925

[b93] Baraban L. . Fuel-free locomotion of janus motors: magnetically induced thermophoresis. ACS Nano 7, 1360–1367 (2013). 23268780 10.1021/nn305726m

[b94] Wheeler M. A. . Genetically targeted magnetic control of the nervous system. Nat. Neurosci. 19, 756–761 (2016). 26950006 10.1038/nn.4265 PMC4846560

[b95] Kilinc D., Dennis C. L. & Lee G. U. Bio-nano-magnetic materials for localized mechanochemical stimulation of cell growth and death. Adv. Mater. 28, 5672–5680 (2016). 26780501 10.1002/adma.201504845 PMC5536250

[b96] Wang N. & Ingber D. E. Probing transmembrane mechanical coupling and cytomechanics using magnetic twisting cytometry. Biochem. Cell Biol. 73, 327–335 (1995). 8703406 10.1139/o95-041

[b97] Cartmell S. H., Dobson J., Verschueren S. B. & El Haj A. J. Development of magnetic particle techniques for long-term culture of bone cells with intermittent mechanical activation. IEEE Trans. Nanobiosci. 1, 92–97 (2002). 10.1109/tnb.2002.806945 16689213

[b98] Muroski M. E. . Controlled payload release by magnetic field triggered neural stem cell destruction for malignant glioma treatment. PLoS ONE 11, 4–15 (2016). 10.1371/journal.pone.0145129 PMC4703386 26734932

[b99] Kim D.-H. . Biofunctionalized magnetic-vortex microdiscs for targeted cancer-cell destruction. Nat. Mater. 9, 165–171 (2010). 19946279 10.1038/nmat2591 PMC2810356

[b100] Hein M. A. . Fabrication of bioinspired inorganic nanocilia sensors. IEEE Trans. Magn. 49, 191–196 (2013).

[b101] Brataas A., Kent A. D. & Ohno H. Current-induced torques in magnetic materials. Nat. Mater. 11, 372–381 (2012). 22522637 10.1038/nmat3311

[b102] Locatelli N., Cros V. & Grollier J. Spin-torque building blocks. Nat. Mater. 13, 11–20 (2014). 24343514 10.1038/nmat3823

[b103] Parkin S. S. P., Hayashi M. & Thomas L. Magnetic domain-wall racetrack memory. Science 320, 190–194 (2008) ***A review of the development of racetrack memory, including the proposal of a vertical racetrack***. 18403702 10.1126/science.1145799

[b104] Ishigaki T. . A multi-level-cell spin-transfer torque memory with series-stacked magnetotunnel junctions. In 2010 Symposium on VLSI Technology 47–48IEEE (2010).

[b105] Kazuo K., Morihiro K. & Kenji T. (eds). *Three-Dimensional Integration of Semiconductors Processing, Materials, and Applications* (Springer, 2015).

[b106] Ono T. H., Miyajima K., Shigeto K., Mibu N. & Hosoito T. S. Propagation of a magnetic domain wall in a submicrometer magnetic wire. Science 284, 468–470 (1999) ***This work shows domain wall conduit behaviour in nanowires***. 10205050 10.1126/science.284.5413.468

[b107] Chumak A. V., Vasyuchka V. I., Serga A. A. & Hillebrands B. Magnon spintronics. Nat. Phys. 11, 453–461 (2015).

[b108] Allwood D. A. . Magnetic domain-wall logic. Science 309, 1688–1692 (2005) ***This paper demonstrates domain wall logic based on magnetic nanowires***. 16151002 10.1126/science.1108813

[b109] Lequeux S. . A magnetic synapse: multilevel spin-torque memristor with perpendicular anisotropy. Sci. Rep. 6, 31510 (2016). 27539144 10.1038/srep31510 PMC4990964

[b110] Von Neumann J., Churchland P. M. & Churchland P. S. The Computer And The Brain Yale University Press (2000).

[b111] Bauer G. E. W., Saitoh E. & van Wees B. J. Spin caloritronics. Nat. Mater. 11, 391–399 (2012). 22522639 10.1038/nmat3301

[b112] Fusil S., Garcia V., Barthélémy A. & Bibes M. Magnetoelectric devices for spintronics. Annu. Rev. Mater. Res. 44, 91–116 (2014).

[b113] Xu S. . Materials science. Assembly of micro/nanomaterials into complex, three-dimensional architectures by compressive buckling. Science 347, 154–159 (2015). 25574018 10.1126/science.1260960

[b114] Fisher J. S., Kottke P. A., Kim S. & Fedorov A. G. Rapid electron beam writing of topologically complex 3D nanostructures using liquid phase precursor. Nano Lett. 15, 8385–8391 (2015). 26561872 10.1021/acs.nanolett.5b04225

[b115] Fix T. . Electric-field control of ferromagnetism in a nanocomposite via a ZnO phase. Nano Lett. 13, 5886–5890 (2013). 24283467 10.1021/nl402775h

[b116] Rusz J., Idrobo J.-C. & Bhowmick S. Achieving atomic resolution magnetic dichroism by controlling the phase symmetry of an electron probe. Phys. Rev. Lett. 113, 145501 (2014). 25325649 10.1103/PhysRevLett.113.145501

[b117] Guzzinati G. . Prospects for versatile phase manipulation in the TEM: beyond aberration correction. Ultramicroscopy 151, 85–93 (2015). 25455416 10.1016/j.ultramic.2014.10.007

[b118] Lutman A. A. . Polarization control in an X-ray free-electron laser. Nat. Photonics 10, 468–472 (2016).

[b119] Park H. S., Baskin J. S. & Zewail A. H. 4D Lorentz electron microscopy imaging: magnetic domain wall nucleation, reversal, and wave velocity. Nano Lett. 10, 3796–3803 (2010). 20735136 10.1021/nl102861e

[b120] Gao F. & Gu Z. Nano-soldering of magnetically aligned three-dimensional nanowire networks. Nanotechnology 21, 115604 (2010). 20179331 10.1088/0957-4484/21/11/115604

[b121] Yang S.-H., Ryu K.-S. & Parkin S. Domain-wall velocities of up to 750ms(-1) driven by exchange-coupling torque in synthetic antiferromagnets. Nat. Nanotechnol. 10, 221–226 (2015). 25705867 10.1038/nnano.2014.324

[b122] Braun H.-B. Topological effects in nanomagnetism: from superparamagnetism to chiral quantum solitons. Adv. Phys. 61, 1–116 (2012).

[b123] Markov I. L. Limits on fundamental limits to computation. Nature 512, 147–154 (2014). 25119233 10.1038/nature13570

[b124] Wang R. F. . Artificial ‘spin ice' in a geometrically frustrated lattice of nanoscale ferromagnetic islands. Nature 439, 303–306 (2006). 16421565 10.1038/nature04447

[b125] Donnelly C. . Element-specific X-ray phase tomography of 3D structures at the nanoscale. Phys. Rev. Lett. 114, 115501 (2015). 25839287 10.1103/PhysRevLett.114.115501

[b126] Tottori S. . Magnetic helical micromachines: fabrication, controlled swimming, and cargo transport. Adv. Mater. 24, 811–816 (2012). 22213276 10.1002/adma.201103818

[b127] Albrecht M. . Magnetic multilayers on nanospheres. Nat. Mater. 4, 203–206 (2005). 15711553 10.1038/nmat1324

[b128] Streubel R. . Equilibrium magnetic states in individual hemispherical permalloy caps. Appl. Phys. Lett. 101, 132419 (2012).

[b129] Eslami S. . Chiral nanomagnets. ACS Photonics 1, 1231–1236 (2014).

[b130] Gibbs J. G. . Nanohelices by shadow growth. Nanoscale 6, 9457–9466 (2014). 24841858 10.1039/c4nr00403e

[b131] Yannopapas V. & Vanakaras A. G. Strong magnetochiral dichroism in suspensions of magnetoplasmonic nanohelices. ACS Photonics 2, 1030–1038 (2015).

[b132] Schmidt O. G. & Eberl K. Nanotechnology: thin solid films roll up into nanotubes. Nature 410, 168–168 (2001) ***This brief communication describes the development of rolled-up nanotechnology***. 11242068 10.1038/35065525

[b133] Lara P. . in Advances in Organometallic Chemistry and Catalysis: the Silver/Gold Jubilee International Conference on Organometallic Chemistry Celebratory Book ed. Pombeiro A. J. L. Ch. 31, Wiley (2014).

[b134] Anagnostopoulou E. . Dense arrays of cobalt nanorods as rare-earth free permanent magnets. Nanoscale 8, 4020–4029 (2016). 26817959 10.1039/c5nr07143g

[b135] Liakakos N. . Solution epitaxial growth of cobalt nanowires on crystalline substrates for data storage densities beyond 1 Tbit/in^2^. Nano Lett. 14, 3481–3486 (2014). 24828234 10.1021/nl501018z

[b136] Vivas L. G., Escrig J., Trabada D. G., Badini-Confalonieri G. A. & Väzquez M. Magnetic anisotropy in ordered textured Co nanowires. Appl. Phys. Lett. 100, 252405 (2012).

[b137] Zierold R. . Magnetic, multilayered nanotubes of low aspect ratios for liquid suspensions. Adv. Funct. Mater. 21, 226–232 (2011).

[b138] Ozel T., Bourret G. R. & Mirkin C. A. Coaxial lithography. Nat. Nanotechnol. 10, 319–324 (2015). 25799520 10.1038/nnano.2015.33

[b139] Lee J. H. . Iron–gold barcode nanowires. Angew. Chem. Int Ed. 46, 3663–3667 (2007). 10.1002/anie.200605136 17407120

[b140] Piraux L. . Template-grown NiFe/Cu/NiFe nanowires for spin transfer devices. Nano Lett. 7, 2563–2567 (2007). 17715984 10.1021/nl070263s

[b141] Huang X., Tan L., Cho H. & Stadler B. J. H. Magnetoresistance and spin transfer torque in electrodeposited Co/Cu multilayered nanowire arrays with small diameters. J. Appl. Phys. 105, 07D128 (2009).

[b142] Sousa C. T. . Nanoporous alumina as templates for multifunctional applications. Appl. Phys. Rev. 1, 031102 (2014).

[b143] Lee W. . Structural engineering of nanoporous anodic aluminium oxide by pulse anodization of aluminium. Nat. Nanotechnol. 3, 234–239 (2008). 18654508 10.1038/nnano.2008.54

[b144] Ross C. A., Berggren K. K., Cheng J. Y., Jung Y. S. & Chang J.-B. Three-dimensional nanofabrication by block copolymer self-assembly. Adv. Mater. 26, 4386–4396 (2014). 24706521 10.1002/adma.201400386

[b145] Mistonov A. A. . Three-dimensional artificial spin ice in nanostructured Co on an inverse opal-like lattice. Phys. Rev. B 87, 220408 (2013).

[b146] Hsueh H.-Y. . Nanoporous gyroid nickel from block copolymer templates via electroless plating. Adv. Mater. 23, 3041–3046 (2011). 21598314 10.1002/adma.201100883

[b147] Utke I., Hoffmann P. & Melngailis J. Gas-assisted focused electron beam and ion beam processing and fabrication. J. Vac. Sci. Technol. B Microelectron. Nanom. Struct. 26, 1197–1276 (2008).

[b148] Fernández-Pacheco A., De Teresa J. M., Córdoba R. & Ibarra M. R. Magnetotransport properties of high-quality cobalt nanowires grown by focused-electron-beam-induced deposition. J. Phys. D Appl. Phys. 42, 055005 (2009) ***This paper reports the 3D nano-printing of ferromagnetic nanostructures with purities above 90%***.

[b149] De Teresa J. M. . Review of magnetic nanostructures grown by focused electron beam induced deposition (FEBID). J. Phys. D Appl. Phys. 49, 243003 (2016).

[b150] Bernau L., Gabureac M., Erni R. & Utke I. Tunable nanosynthesis of composite materials by electron-impact reaction. Angew. Chem. 122, 9064–9068 (2010). 10.1002/anie.201004220 20936609

[b151] Porrati F. . Direct writing of CoFe alloy nanostructures by focused electron beam induced deposition from a heteronuclear precursor. Nanotechnology 26, 475701 (2015). 26535785 10.1088/0957-4484/26/47/475701

[b152] Costanzi B. N., Riazanova A. V., Dan Dahlberg E. & Belova L. M. In situ manufacture of magnetic tunnel junctions by a direct-write process. Appl. Phys. Lett. 104, 222401 (2014).

[b153] Utke I., Hoffmann P., Berger R. & Scandella L. High-resolution magnetic Co supertips grown by a focused electron beam. Appl. Phys. Lett. 80, 4792–4794 (2002).

[b154] Fernández-Pacheco A. . Three dimensional magnetic nanowires grown by focused electron-beam induced deposition. Sci. Rep. 3, 1492 (2013) ***This work demonstrates the magneto-optical detection of magnetic nanowires in a 3D configuration***. 23512183 10.1038/srep01492 PMC3603301

[b155] Takeguchi M., Shimojo M., Che R. & Furuya K. Fabrication of a nano-magnet on a piezo-driven tip in a TEM sample holder. J. Mater. Sci. 41, 2627–2630 (2006).

[b156] Lau Y. M., Chee P. C., Thong J. T. L. & Ng V. Properties and applications of cobalt-based material produced by electron-beam-induced deposition. J. Vac. Sci. Technol. A Vac. Surf. Film 20, 1295–1302 (2002).

[b157] Esposito M. . Nanoscale 3D chiral plasmonic helices with circular dichroism at visible frequencies. ACS Photonics 2, 105–114 (2015).

[b158] Botman A., Mulders J. J. L. & Hagen C. W. Creating pure nanostructures from electron-beam-induced deposition using purification techniques: a technology perspective. Nanotechnology 20, 372001 (2009). 19706953 10.1088/0957-4484/20/37/372001

[b159] Plank H. . Electron-beam-assisted oxygen purification at low temperatures for electron-beam-induced pt deposits: towards pure and high-fidelity nanostructures. ACS Appl. Mater. Interfaces 6, 1018–1024 (2014). 24377304 10.1021/am4045458

[b160] Roberts N. A., Fowlkes J. D., Magel G. A. & Rack P. D. Enhanced material purity and resolution via synchronized laser assisted electron beam induced deposition of platinum. Nanoscale 5, 408–415 (2013). 23184056 10.1039/c2nr33014h

[b161] Fowlkes J. D. Simulation guided 3D nanomanufacturing via focused electron beam induced deposition. ACS Nano 10, 6163–6172 (2016) ***This article presents the 3D nano-printing of complex nanostructures making use of modelling based on Monte Carlo and continuum model methods***. 27284689 10.1021/acsnano.6b02108

[b162] Wernsdorfer W. . Nucleation of magnetization reversal in individual nanosized nickel wires. Phys. Rev. Lett. 77, 1873–1876 (1996). 10063193 10.1103/PhysRevLett.77.1873

[b163] Allwood D. a., Xiong G., Cooke M. D. & Cowburn R. P. Magneto-optical Kerr effect analysis of magnetic nanostructures. J. Phys. D Appl. Phys. 36, 2175–2182 (2003).

[b164] Jaafar M. . Hysteresis loops of individual Co nanostripes measured by magnetic force microscopy. Nanoscale Res. Lett. 6, 407 (2011). 21711935 10.1186/1556-276X-6-407 PMC3211502

[b165] Weber D. P. . Cantilever magnetometry of individual Ni nanotubes. Nano Lett. 12, 6139–6144 (2012). 23134122 10.1021/nl302950u

[b166] Buchter A. . Reversal mechanism of an individual Ni nanotube simultaneously studied by torque and SQUID magnetometry. Phys. Rev. Lett. 111, 067202 (2013). 23971606 10.1103/PhysRevLett.111.067202

[b167] Banerjee P. . Magnetization reversal in an individual 25 nm iron-filled carbon nanotube. Appl. Phys. Lett. 96, 252505 (2010).

[b168] Fitzsimmons M. R. & Schuller I. K. Neutron scattering—the key characterization tool for nanostructured magnetic materials. J. Magn. Magn. Mater. 350, 199–208 (2014).

[b169] Adams T. . Long-wavelength helimagnetic order and skyrmion lattice phase in Cu 2 OSeO 3. Phys. Rev. Lett. 108, 237204 (2012). 23003986 10.1103/PhysRevLett.108.237204

[b170] Tokunaga Y. . A new class of chiral materials hosting magnetic skyrmions beyond room temperature. Nat. Commun. 6, 7638 (2015). 26134284 10.1038/ncomms8638 PMC4506512

[b171] Manke I. . Three-dimensional imaging of magnetic domains. Nat. Commun. 1, 125 (2010). 21119638 10.1038/ncomms1125

[b172] Maurer T. . Ordered arrays of magnetic nanowires investigated by polarized small-angle neutron scattering. Phys. Rev. B 89, 184423 (2014).

[b173] Fischer P. X-ray imaging of magnetic structures. IEEE Trans. Magn. 51, 1–31 (2015).26203196

[b174] Reyes D., Biziere N., Warot-Fonrose B., Wade T. & Gatel C. Magnetic configurations in Co/Cu multilayered nanowires: evidence of structural and magnetic interplay. Nano Lett. 16, 1230–1236 (2016). 26783831 10.1021/acs.nanolett.5b04553

[b175] Yu X. Z. . Real-space observation of a two-dimensional skyrmion crystal. Nature 465, 901–904 (2010). 20559382 10.1038/nature09124

[b176] Zhao X. . Direct imaging of magnetic field-driven transitions of skyrmion cluster states in FeGe nanodisks. Proc. Natl Acad. Sci. USA 113, 4918–4923 (2016). 27051067 10.1073/pnas.1600197113 PMC4983818

[b177] Ivanov Y. P., Chuvilin A., Lopatin S. & Kosel J. Modulated magnetic nanowires for controlling domain wall motion: toward 3D magnetic memories. ACS Nano 10, 5326–5332 (2016). 27138460 10.1021/acsnano.6b01337

[b178] Midgley P. A. & Dunin-Borkowski R. E. Electron tomography and holography in materials science. Nat. Mater. 8, 271–280 (2009). 19308086 10.1038/nmat2406

[b179] Phatak C., Tanase M., Petford-Long A. K. & De Graef M. Determination of magnetic vortex polarity from a single Lorentz Fresnel image. Ultramicroscopy 109, 264–267 (2009). 19110377 10.1016/j.ultramic.2008.11.003

[b180] Wolf D. . 3D magnetic induction maps of nanoscale materials revealed by electron holographic tomography. Chem. Mater. 27, 6771–6778 (2015). 27182110 10.1021/acs.chemmater.5b02723 PMC4862384

[b181] Simon P. . Synthesis and three-dimensional magnetic field mapping of Co_2_ FeGa Heusler nanowires at 5 nm resolution. Nano Lett. 16, 114–120 (2016). 26674206 10.1021/acs.nanolett.5b03102

[b182] Phatak C. . Visualization of the magnetic structure of sculpted three-dimensional cobalt nanospirals. Nano Lett. 14, 759–764 (2014). 24444002 10.1021/nl404071u

[b183] Phatak C. . Quantitative 3D electromagnetic field determination of 1D nanostructures from single projection. Ultramicroscopy 164, 24–30 (2016). 26998702 10.1016/j.ultramic.2016.03.005

[b184] Phatak C., Petford-Long A. K. & De Graef M. Three-dimensional study of the vector potential of magnetic structures. Phys. Rev. Lett. 104, 253901 (2010) ***This paper demonstrates three-dimensional magnetic vectorial tomography using electron microscopy***. 20867379 10.1103/PhysRevLett.104.253901

[b185] Tanigaki T. . Three-dimensional observation of magnetic vortex cores in stacked ferromagnetic discs. Nano Lett. 15, 1309–1314 (2015). 25594686 10.1021/nl504473a

[b186] Kimling J. . Photoemission electron microscopy of three-dimensional magnetization configurations in core-shell nanostructures. Phys. Rev. B 84, 174406 (2011).

[b187] Streubel R. . Retrieving spin textures on curved magnetic thin films with full-field soft X-ray microscopies. Nat. Commun. 6, 7612 (2015) ***This work demonstrates three-dimensonal magnetic vectorial tomography using X-ray microscopy***. 26139445 10.1038/ncomms8612 PMC4506513

[b188] Streubel R. . Imaging of buried 3D magnetic rolled-up nanomembranes. Nano Lett. 14, 3981–3986 (2014). 24849571 10.1021/nl501333h PMC4096489

[b189] Blanco-Roldán C. . Nanoscale imaging of buried topological defects with quantitative X-ray magnetic microscopy. Nat. Commun. 6, 8196 (2015). 26337838 10.1038/ncomms9196 PMC4569793

[b190] Hertel R. . Three-dimensional magnetic-flux-closure patterns in mesoscopic Fe islands. Phys. Rev. B 72, 214409 (2005).

[b191] Kakay A., Westphal E. & Hertel R. Speedup of FEM micromagnetic simulations with graphical processing units. IEEE Trans. Magn. 46, 2303–2306 (2010).

[b192] Shaojing L., Livshitz B. & Lomakin V. Graphics processing unit accelerated *O*(*N*) micromagnetic solver. IEEE Trans. Magn. 46, 2373–2375 (2010).

[b193] Sato T. & Nakatani Y. Fast micromagnetic simulation of vortex core motion by GPU. J. Magn. Soc. Jpn 35, 163–170 (2011).

[b194] Kittel C. Physical theory of ferromagnetic domains. Rev. Mod. Phys. 21, 541–583 (1949).

[b195] Andreas C., Gliga S. & Hertel R. Numerical micromagnetism of strong inhomogeneities. J. Magn. Magn. Mater. 362, 7–13 (2014).

[b196] Evans R. F. L. . Atomistic spin model simulations of magnetic nanomaterials. J. Phys. Condens. Matter 26, 103202 (2014). 24552692 10.1088/0953-8984/26/10/103202

[b197] Bergqvist L., Taroni A., Bergman A., Etz C. & Eriksson O. Atomistic spin dynamics of low-dimensional magnets. Phys. Rev. B 87, 144401 (2013).

